# An Adaptive Background Subtraction Method Based on Kernel Density Estimation

**DOI:** 10.3390/s120912279

**Published:** 2012-09-07

**Authors:** Jeisung Lee, Mignon Park

**Affiliations:** School of Electrical and Electronic Engineering, Yonsei University, 134 Shinchon-Dong, Seodaemun-Gu, Seoul 120-749, Korea; E-Mail: leejaisung@yonsei.ac.kr

**Keywords:** background subtraction, kernel density estimation, video surveillance, adaptive background estimation

## Abstract

In this paper, a pixel-based background modeling method, which uses nonparametric kernel density estimation, is proposed. To reduce the burden of image storage, we modify the original KDE method by using the first frame to initialize it and update it subsequently at every frame by controlling the learning rate according to the situations. We apply an adaptive threshold method based on image changes to effectively subtract the dynamic backgrounds. The devised scheme allows the proposed method to automatically adapt to various environments and effectively extract the foreground. The method presented here exhibits good performance and is suitable for dynamic background environments. The algorithm is tested on various video sequences and compared with other state-of-the-art background subtraction methods so as to verify its performance.

## 1. Introduction

One of the most important aspects of an intelligent vision surveillance system is background subtraction, which is used as a preprocessing step for object detection and tracking in vision systems. Usually, every pixel is searched and compared step-by-step with a predefined object dataset so as to detect or track an object. However, searching every pixel requires a high computational time and thus, a background subtraction method is generally used to reduce the searching region and improve computational performance. Background subtraction is also used in human-computer interactions (HCI) as a preprocessing step to reduce computational cost. As such, background subtraction is an important subject in the field of computer vision. Since background modeling significantly affects the performance of the overall vision system, it is important to employ a good background subtraction method. However, many challenges are associated with background modeling.

- Dynamic backgrounds: The background is generally non-static (e.g., waving trees, swaying curtains, escalators, rippling water surfaces, *etc.*) and thus, it should be removed to extract the foreground.
- Gradual illumination changes: These are caused by either sunlight changes as time elapses or by the sun being covered by clouds.
- Sudden illumination changes: Light can sometimes be switched on or off in indoor environments. This can significantly change the background. Thus, a modeled background should quickly adapt to environmental changes.
- Moved object: A background should be changed by a moved object. If someone parks a car and the car is not moved for a long period of time, the car should be accepted as part of the background.
- Shadows: Usually, the shadows of moving objects need to be eliminated.

Another challenge is that many moving foregrounds can appear simultaneously with the above non-static problems. Therefore, background modeling methods should intelligently overcome such issues.

There are three representative approaches for background subtraction methods. First, pixel-based methods extract foregrounds using each pixel independently. Such approaches do not consider the relationships among the surrounding pixels. One of the most commonly used pixel-based methods is Gaussian modeling. Wren *et al.* [1] and Horprasert *et al.* [2] proposed a single Gaussian model to model the background. However, since the background is usually non-static, the use of a single Gaussian model is not sufficient to remove the background. The Mixture of Gaussians (MOG) technique [3,4] is more useful for modeling the background than the single Gaussian method. The MOG scheme overcomes the drawback of the single Gaussian model by assuming the existence of a dynamic background and employing a multi-Gaussian model. Chiu *et al.* [5] proposed a probabilistic approach and foreground extraction method that suitably extracts the foreground for each image environment using the color distribution. This algorithm is very fast and robust; it can extract a robust background model even if many moving objects are present during the training time. However, the algorithm does not consider a dynamic background environment and thus, it only exhibits good performance for a static background. Kim *et al.* [6] proposed a codebook model. Sample background values at each pixel are quantized into codebooks that represent a compressed form of the background model. Codewords not appearing for a long period of time in the sequence are eliminated from the codebook model and new images that have appeared for some time are quantized into codebooks. While this algorithm is not especially fast, it was very effective for dynamic backgrounds. Maddalena *et al.* [7] proposed an approach based on a self-organizing feature map that is widely applied in human image processing and more generally implemented in cognitive science. While the algorithm exhibited good performance and faster speeds than the codebook scheme, many parameters must be manually selected according to the video environment. To solve the drawbacks of manually selecting parameters in each environment, non-parametric approach methods were proposed by Elgammal *et al.* [8], Lanasi [9] and Park *et al.* [10]. The latter [10] used the Bayesian rule with the kernel density estimation (KDE) method [8] and applied histogram approximation to decrease the computational cost. Palmen *et al.* [11] proposed a recursive density estimation (RDE) method. They applied Cauchy-type function of the KDE model to modeling backgrounds. This method does not require much memory space and has a faster speed (shorter training time) than the original KDE method. However, the limitation of RDE method is that it's simply based on tracking approach. If the background in a pixel has waving sequences in a large scale, there may have some possibility that the algorithm misclassify the foreground as background. For instance, a foreground appeared in a waving-tree pixel sequence.

Another group of background subtraction techniques are the block-based methods. Among these techniques, the Markov random field framework was used by Reddy for background estimation [12]. The method was very effective for background estimation, but was more appropriate for use in an indoor environment. In addition, the method only estimated a static background model and was not used to extract foreground samples. Matsuyama *et al.* [13] employed the normalized vector distance (NVD) in their research, where the foreground was extracted by comparing correlations among neighboring blocks. Mason *et al.* [14] used edge histograms for pixel blocks in order to model the background, while Monnet *et al.* [15] proposed an online auto-regressive model and employed incremental principal component analysis (PCA) to capture and predict the behaviors of waving trees, beaches, and escalators. Chen *et al.* [16] suggested a hierarchical method using block-based and pixel-based MOG schemes. The method exhibited better performance than MOG, but the complexity and computational cost of the algorithm were excessively high. Cuo *et al.* [17] proposed a hierarchical method based on the codebook algorithm [6]. In the block-based stage, the algorithm removes most of the background. A pixel-based step based on the codebook is then adopted to enhance the precision. The method exhibited good performance and was faster than the original codebook scheme. However, if the foreground is relatively small when compared to the block size, it can be deleted as the background by the block-based approach. Varcheie *et al.* [18] combined a region-based method based on color histograms and texture information with the Gaussian mixture model to model the background and detection motion. The method exhibited better performance than the state-of-art background subtraction methods, but the complexity was excessively high.

The third class of background subtraction approaches are the texture-based methods. Heikkila *et al.* [19] used an adaptive local binary pattern (LBP) to extract features from an image. Binary patterns were computed by comparing neighboring pixel values with a center pixel. Specifically, binary patterns were calculated for a circular region around a given center pixel. Such binary patterns were used as a feature to model the background. This method can also be employed to solve non-static background problems, but difficulties in distinguishing areas of uniform texture are encountered. The resulting segmentation is also limited to a resolution of around the circle radius because the texture is calculated over a circular region around the circle radius.

Many background subtraction algorithms have also been proposed. Each algorithm has produced effective foreground extraction results in a limited environment. However, more robust and faster algorithms are constantly required because, as a preprocessing step, exact foreground extraction produces good results in terms of detecting or tracking an object. In this paper, we used a pixel-based method since it is simpler and faster than block-based or hierarchical methods and yields more precise results. Specifically, we propose an adaptive background subtraction method based on kernel density estimation in a pixel-based method. Through the use of kernel density estimation, we can adaptively devise a probabilistic background model in each environment. The proposed method can automatically adapt to various environments and stochastically delete non-background information or add new-background values. In addition, the scheme can quickly adapt to sudden or gradual illumination changes. In Section 2, we present the proposed method and background modeling scheme. In Section 3, well-known sequences are used to compare the performance of the proposed method to that of other state-of-art methods. Finally, conclusions are presented in Section 4.

## 2. Background Subtraction Method

### 2.1. Probability Background Model Using Kernel Density Estimation

Backgrounds are generally non-static with many dynamic factors such as waving trees, rippling water, and illumination changes. Various attempts have been made to overcome these problems. One of the most useful methods is the MOG method, but MOG parameters such as the number of Gaussian models and variances should be manually selected and thus, it takes too much time to initialize the background model with the expectation maximization (EM) algorithm in every pixel independently.

In this paper, we used the kernel density estimation (KDE) method [8], a non-parametric approach that can effectively adapt to a dynamic background. In each pixel, the KDE is calculated by the following equation at time index *t*:

(1) $$p(x)=\frac{1}{n}\sum_{t=1}^{n}K(x-x_{t})$$

where n is the number of total observed frames and *x_t_* is the observed value at time index t. *p*(*x*) is an average of normal densities centered at the sample *x*. The kernel function *K*(*x*) should satisfy the following conditions: ∫*K*(*x*)*dx* = 1, ∫*xK*(*x*)*dx* = 0, and *K*(*x*) > 0.Typically, the normal distribution *N*(0,1) is used as the kernel function. In research conducted by Park *et al.* [10], many frames were collected before estimating the Gaussian background model and thus, a large amount of memory space was required. To overcome this drawback, we modify the original KDE method and propose a scheme that uses the first frame to initialize the KDE background model. In the first frame, most of the pixels represent background, and there are foregrounds in some other pixels. Even if we used the first frame to initialize background model, foreground information will be reduced and remain only background information by updating process because background values are more frequent than foreground values at the pixel level. The KDE Gaussian model is subsequently updated at every frame by controlling the learning rate according to the situation. The probability *p_t_*(*x*) is based on each pixel and may be expressed as:

(2) $$p_{t}(x)={\hat{p}}_{t-1}(x)+\frac{1}{G_{t}\sqrt{2\pi \sigma^{2}}}\exp\left(-\frac{1}{2}{\left(\frac{x-x_{t}}{\sigma }\right)}^{2}\right)$$

Each pixel has a probability model. The probability obtained by the KDE method is added to the prior probability density at every frame. In Equation (2)*G_t_* is used as the learning rate at time *t* and can be changed depending on factors such as time and illumination changes. Since the probability should satisfy ∫*p_t_*(*x*)*dx* = 1, *p_t_*(*x*) is normalized as follows:

(3) $${\hat{p}}_{t}(x)=p_{t}(x)/\sum_{x=0}^{N}p_{t}(x)$$

where *p_t_*(*x*) is a normal density at the sample *x* and at time index *t.* ^*p_t_(x)* is a normalized normal density and *N* is the total number of samples.

A new probability background model is obtained through the above process. This updating method improves memory effectiveness because it does not require many images to be saved to initialize the probability background model. The updating method automatically reduces the probability of unimportant backgrounds that do not appear over a long period of time by adding an additional probability and performing a normalization step. For example, when a car parked for a long period of time moves or disappears, the proposed method continually updates the environment. Consequently, new background information appears and the prior unimportant background probability associated with the car is automatically lowered by updating the background model. We used *G_t_* as a parameter to control the learning rate. If *G_t_* is increased, new information is slowly learned and prior information slowly disappears. If *G_t_* is decreased, the algorithm quickly adapts to the environment and quickly deletes old information. In the initial stage, the background model should quickly adapt to the new environment and, as time elapses, the background should have a stable updating process. For this reason, *G_t_* was used as a sigmoid function which can expressed as follows:

(4) $$G_{t}=\text{Gain}\times \frac{2}{1+\exp(-(\text{cnt}-\beta )/\lambda )}$$

**Figure 1.**
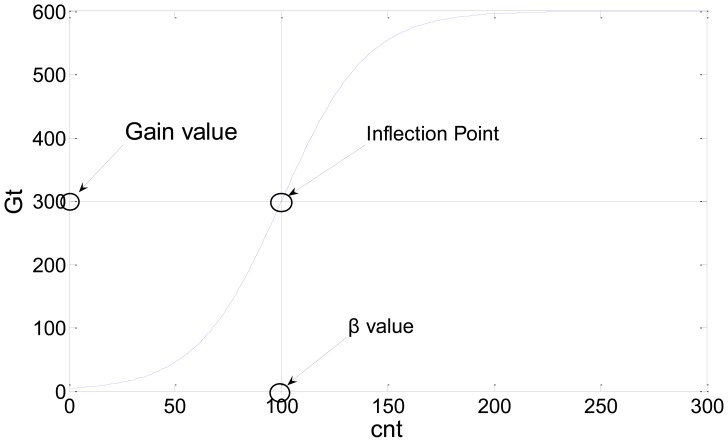
Example of the value of *G_t_* over time.

The value of *G_t_* over time is shown in Figure 1.

In Equation (4), the value of *cnt* increases proportionally with respect to time and can be used to initialize the background by initializing or control the learning rate through the environment by initializing the value of *cnt*. The inflection point is controlled by *β* and *Gain*, while the gradient can be changed by *λ*. The learning rate of the proposed method is affected by the *Gain* parameter. If the *Gain* parameter increases, the learning rate of the algorithm will decrease and *vice versa*. In our experiments, *β* was set to 100, the *Gain* was 300, and *λ* is 20.

**Figure 2.**
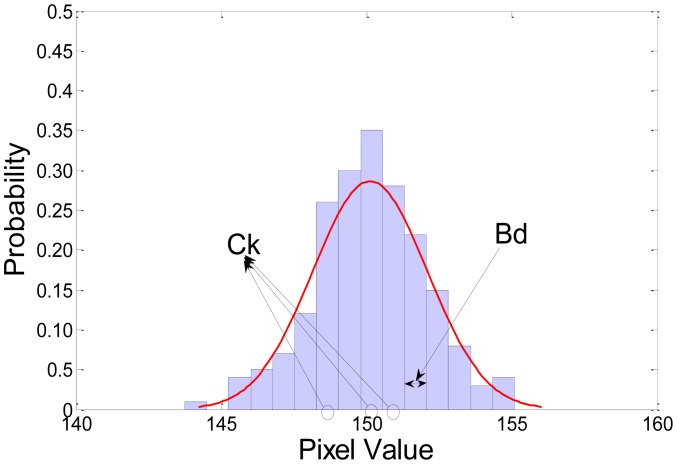
Gaussian probability and an example of histogram approximation; *B_d_* is the width of the histograms in dimension *d* and *C_k_* is the center of each histogram.

A few of the problems associated with the non-parametric kernel density estimation approach are the undesirably long processing time and the large memory requirement. We can reduce the complexity and memory requirement using histogram approximation. The Gaussian probability and an example of histogram approximation are shown in Figure 2. In the figure, *B_d_* is the width of the histograms along dimension *d*, *C_k_* is the center of each histogram, and *k* is the histogram number. The parameter *B_d_* can be calculated according to the following equation:

(5) $$\begin{array}{cc} B_{d}=\frac{\max(x^{d})-\min(x^{d})}{N_{d}} & \;d=1,2,3 \end{array}$$

where *N_d_* represents the number of bins for each dimension *d* and *x^d^* is the value of a pixel in the *d* dimension. A general image has three dimensions: R, G, and B. Thus, the range of *d* is 1 ≤ *d* ≤ 3. The change in the kernel density estimation by histogram approximation may be expressed as follows:

(6) $$\begin{array}{cc} {p_{t}}^{d}(C_{k})={{\hat{p}}_{t-1}}^{d}(C_{k})+\frac{1}{G_{t}\sqrt{2\pi {\left(B_{d}/2\right)}^{2}}}\exp\left(-\frac{1}{2}{\left(\frac{C_{k}-x_{t}^{d}}{B_{d}/2}\right)}^{2}\right) & \;k=1,2\ldots .N_{d} \end{array}d=1,2,3$$

A normalization method was employed in this work since the probability *p_t_^d^*(*C_k_*) should also satisfy the following condition: ∫*p_t_^d^(C_k_)dC_k_* = 1:

(7) $$\begin{array}{cc} {{\hat{p}}_{t}}^{d}(C_{k})={p_{t}}^{d}(C_{k})/\sum_{k=0}^{N_{d}}{p_{t}}^{d}(C_{k}) & \;d=1,2,3 \end{array}$$

where *p_t_^d^*(*C_k_*) is a normal density at the sample *C_k_* and at time index *t. ^ˆ^p_t_^d^*(*C_k_*) is a normalized normal density.

Figure 2 shows an example of 1-D kernel density estimation and histogram approximation. The multidimensional histogram will meet some problems with the increasing memory burden and the complexity of it. To solve these problems, we used three separate histograms according to each dimension; hue, saturation, and illumination.

To reduce the complexity, by taking the integer part after dividing the input with the width of the bin, we may directly find the bin number which the current input belongs to. The *floor* function means that *floor*(A) rounds the elements of A to the nearest integers less than or equal to A:

(8) $$k=\text{floor}({x_{t}}^{d}/B_{d})$$

where *x_t_^d^* is input value, *B_d_* is the width of the bin, and the *k* = 0,2,3,…*N_d_* -1.

For instance, if the input sequences have values in [0 255] and take a bin width of *B_d_* = 4, the bin numbers *k* of the histogram have values in [0 63]. If the input value is 150, we may find the bin number using the Equation (8), *k* = *floor*(150/4) = 37. So the input belongs to the 37th histogram. By using this method, we avoided to search the bins one by one which means a reduction to the complexity.

**Figure 3.**
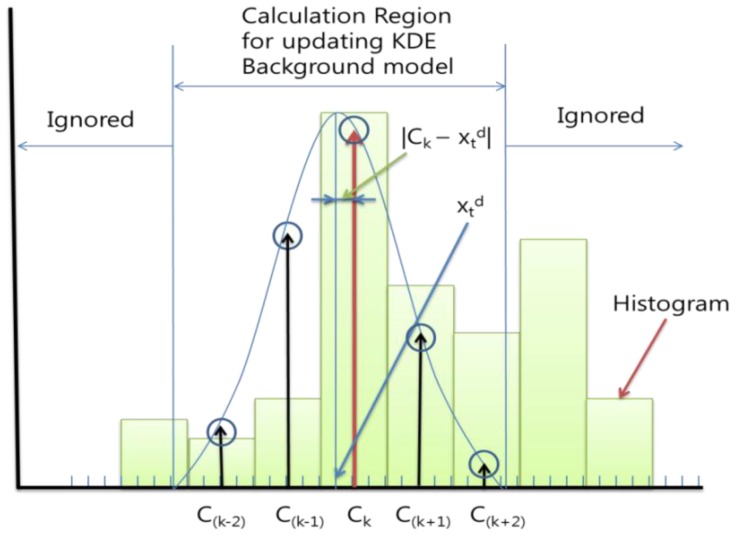
An example of how the histogram was updated.

To update the probability histogram, we applied a Gaussian whose mean value is the input as Equation (6). We can ignore the inference which the input gives to the remote bins, since the Gaussian value quickly falls off towards plus/minus infinity. We calculate the probability of the KDE background model not in whole bins but only for the back and forth less than or equal to *B_d_/2* bins. For example, if the closest center of the input *x_t_^d^* was *C_k_* and the *B_d_* was 4, then we only update the background probability histogram *P_t_^d^*(*C_(k_*_−_*_2)_*)∼*P_t_^d^*(*C_(k_*_+_*_2)_*), with the Equation (6) using *C_(k_*_−_*_2)_*∼*C_(k_*_+_*_2)_*. Figure 3 shows an example of this.

When we update the *P_t_^d^*(*C_(k_*_−_*_2)_*)∼*P_t_^d^*(*C_(k_*_+_*_2)_*), we need to find the Gaussian value of each bin. If we calculate the Equation (9), which is a computational component of Equation (6), every time and in every pixel, it takes too much time. So to reduce the computational cost, we previously calculated and saved the Gaussian probability results according to the case of the difference between the closest center *C_k_* and input value *x_t_^d^*:

(9) $${\text{Pre}}_{p}=\frac{1}{\sqrt{2\pi {\left(B_{d}/2\right)}^{2}}}\exp(-\frac{1}{2}{\left(\frac{C_{k}-{x_{t}}^{d}}{B_{d}/2}\right)}^{2})$$

**Figure 4.**
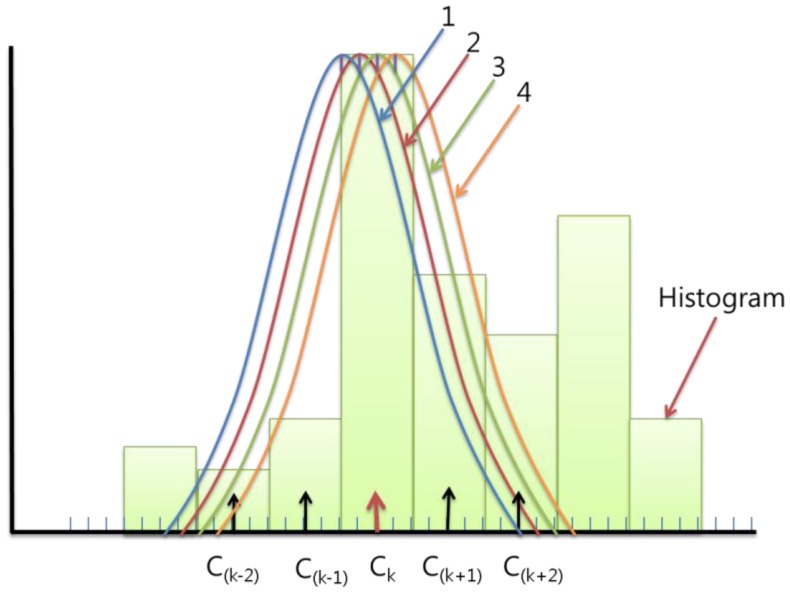
An example of possible Gaussians in a bin.

We consider the case in the example before. If the *B_d_* is 4, there are only four possible Gaussians according to the input value and we only need to save *B_d_* + 1 = 5 values for each Gaussian in each bin as Figures 3 and 4 (the total of the points are *B_d_* × (*B_d_* + 1) = 20). It can cover all the cases we will meet on the updating process. However, the *B_d_* is not usually integer, so if we set *G* = *floor*(*B_d_/2*), we updated the background probability histogram *P_t_^d^*(*C_(k_*_−_*_G)_*)∼*P_t_^d^*(*C_(k_*_+_*_G)_*). The total of the points will be *ceil*(*B_d_*) × (*floor*(*B*_d_) + 1), where *ceil*(A) rounds the elements of A to the nearest integers greater than or equal to A and *floor*(A) rounds the elements of A to the nearest integers less than or equal to A.

If we previously calculated and saved the complex Gaussian computations, we can reduce the computational cost by simply using the saved values in each case when we compute Equation (6).

Most of the background extraction methods used color information, especially RGB color space. However, RGB color is very sensitive to illumination changes, but we can independently analyze both the color itself and illumination changes using HSV color space. Even if the illumination changes significantly, the hue and saturation keep stable. When compared to RGB space, HSV color space is more useful for devising a background model and removing shadows. Therefore, we employed HSV color space to develop the background model. HSV color space is not linear. Hue space does not have linear values, but values repeat periodically. So, we have to consider it during updating the probability histogram. For example, if the bins of the histogram has values in [0 63] and the *B_d_* is 4, we will update the bins of the histogram from *C*_(_*_k_*_−_*_2_*_)_ to *C*_(_*_k_*_+_*_2_*_)_. If the *k* is 10, then we will update the bins from 8 to 12. However, if the *k* is 63, then we will update the bins of 61, 62, 63, 0, and 1.

### 2.2. Foreground Extraction and Background Update

A background subtraction algorithm is composed of two steps: background modeling and background updating. Most background subtraction algorithms collect image frames and use the collected images to generate the background model. The algorithms then extract the foreground using the background model, which is subsequently updated. However, our proposed method does not require an image collection process to generate the background model. The scheme proposed here updates the background model and extracts the foreground at every frame. In other words, the probability background model is initialized by the first frame and updated by the same process with the initialized background. As time elapses, this method automatically adapts to the environment and extracts the foreground in a more precise manner. The proposed method used the minimum distance value between the current image and the background model to obtain the foreground. We also used the average mean value of the minimum distances to adaptively extract the foreground.

#### 2.2.1. Foreground Detection

The foreground is acquired via the following steps. First, the nearest *C_k_* value is obtained with a new input image in each pixel. Here, *C_k_* is the background histogram centers that have a larger probability than 1/*N_d_*. Next, the minimum distance between *C_k_* and the new input values are calculated in each pixel and in each dimension:

(10) $$\begin{array}{cc} {\text{Dist}}_{d}=\underset{\forall k}{\min}({C_{k}}^{d}-x^{d}),\text{where}\;{\hat{p}}^{d}(C_{k})>1/N_{d} & \;d=1,2,3 \end{array}$$

We can obtain the foreground by comparing *Dist_d_* with *B_d_* as follows:

(11) $$\{\begin{array}{cc} if(\sum_{d=1}^{3}\left(\left|{\text{Dist}}_{d}\right|/(1+{\text{Grad}}_{t-1,d})\right)>\sum_{d=1}^{3}B_{d}\times \gamma ) & \\ \text{then},\text{ForG}=1, & \\ \;{\text{Grad}}_{t,d}=(G_{t}-1)\times {\text{Grad}}_{t-1,d}/G_{t}+w\times \left|{\text{Dist}}_{d}\right|/G_{t} & d=1,2,3\\ \text{Else},\text{ForG}=0, & \\ \;{\text{Grad}}_{t,d}=(G_{t}-1)\times {\text{Grad}}_{t-1,d}/G_{t}+\left|{\text{Dist}}_{d}\right|/G_{t} & d=1,2,3 \end{array}$$

where *ForG* is the result obtained from extracting the foreground or a moving object. A *ForG* value of 1 corresponds to the foreground; otherwise a *ForG* value of 0 corresponds to the background. In Equation (1), *Grad_t,d_* is the average of an absolute of *Dist_d_* at time *t*; it is initialized as 1 and subsequently updated. Any value can be selected as an initial value of *Grad_t,d_* because it will automatically find the proper values as updating the *Grad_t,d_*. The parameter w is a weight to control the speed of adaptation for the environments. If *w* is large, the proposed algorithm quickly adapts to the environment and reduces noise. However, if *w* is too large, the algorithm miscalculates the foreground as the background. *w* is in the range of 0.1 to 0.3. In addition, *γ* is the weight for the threshold. Here, *γ* values in the range of 1 to 1.5 can be employed. When we tested the proposed method, we set *w* to 0.1 and *γ* to 1.We divided the *Dist_d_* by (1 + *Grad_t-1,d_*), where *Grad_t-1,d_* is the average of all *Dist_d_* 's absolute at time *t*-1, and we added one to the *Grad_t-1,d_* to avoid a divergence when the *Dist_d_* was divided by *Grad_t-1,d_*. Even though we got a large *Dist_d_*, if the *Grad_t,d_* is also large, the result of |*Dist_d_*|/(1+*Grad_t-1,d_*) is decreasing. So the pixel also can be covered as background. We also used the width of the histograms *B_d_* in the *d* dimension as a threshold. If the sum of the result of |*Dist_d_*|/(1+*Grad_t-1,d_*) is larger than the sum of the width of the histograms, the pixel is classified to foreground.

#### 2.2.2. Shadow Detection

To remove the shadows of moving objects, we applied a moving cast shadow detection algorithm [20] that proved to be quite accurate and suitable for eliminating shadows. The basic idea is that a cast shadow darkens the background, while the color of the background itself is not changed. Using this principle, we can express the removing shadow algorithm as follows:

(12) $$\left(\rho \leq \frac{x^{v}}{Bg^{v}}\leq \delta \right)\wedge \left(\left|x^{s}-Bg^{s}\right|\leq \tau_{s}\right)\wedge \left(\left|x^{h}-Bg^{h}\right|\leq \tau_{h}\right)$$

where *Bg^h^*, *Bg^s^*, and *Bg^v^* represent the hue, saturation, and illumination components, respectively, of the background pixels with background values that are closest to the input image among background histogram models. *x^v^*, *x^s^*, and *x^h^* represent the hue, saturation, and illumination components of the input video pixels. In Equation (12), The ˆ is an *and* operator. According to this principal, we can remove shadows. In our experiments, values for the parameters were chosen as *ρ* = 0.6, *δ* = 1,*τ_s_* = 0.2, and *τ_h_* = 15.

#### 2.2.3. Adaptation for a Sudden Illumination Change

If the background itself is significantly changed (e.g., suddenly brightened or darkened), fast adaptation is required. We can obtain this effect by initializing the *cnt* value. If the value of *cnt* is initialized, *G_t_* is also initialized and the speed of adaptation for the background increases:

(13) $$\{\begin{array}{l} Mv_{t}=(G_{t}-1)\times Mv_{t-1}/G_{t}+\underset{\forall i,j}{\text{mean}}({\text{Dist}}_{v}(i,j))/G_{t}\\ if(|\underset{\forall i,j}{\text{mean}}({\text{Dist}}_{v}(i,j))-Mv_{t}|>T_{v})\;\text{then},\text{cnt}=\beta /2 \end{array}$$

In Equation (13)*T_v_* is a threshold to initialize *cnt*; it is set to 30 in our experiments. *Dist_v_*(*i*, *j*) is a illumination value of current input image at the (*i*, *j*) pixel. *Mv_t_* is an moving average value of *mean_∀i,j_*(*Dist_v_*(*i, j*)).

#### 2.2.4. Summary of the Proposed Algorithm

**Figure 5.**
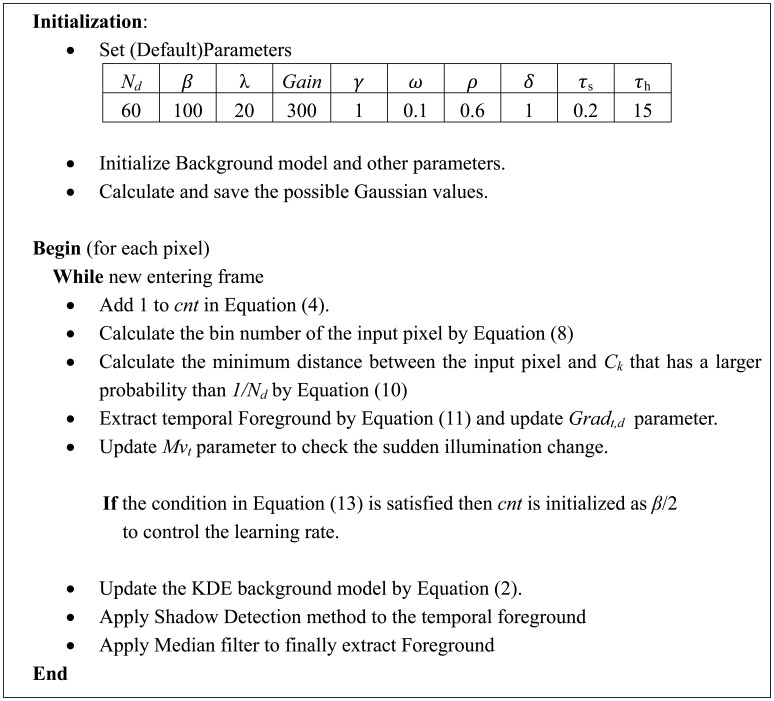
Summary of the proposed algorithm.

Figure 5 shows the summary of the proposed method. In this paper, we tested the proposed method with the default parameter sets in Figure 5, but the parameters can be changed according to the environments.

## 3. Experiments and Analysis

We tested the proposed method with the Li and Wallflower datasets (the Li dataset is available at http://perception.i2r.a-star.edu.sg/bk_model/bk_index.html, while the Wallflower dataset is available at http://research.microsoft.com/en-us/um/people/jckrumm/WallFlower/TestImages.htm). These two datasets are well-known and are often used to test background subtraction algorithms. The datasets were acquired using a fixed camera and thus, they potentially have problematic sequences for background subtraction. To verify the performance of the proposed method, we compared the results obtained with our scheme to those from other state-of-art methods. Before comparing the findings, we applied a median filter to all results so as to reduce noise. During the performance testing, we tried to obtain the best result from the other methods by tuning the relevant parameters.

### 3.1. Performance Measure Method

Three measures were used to evaluate the performance of the proposed method: recall, precision, and F-measure. Recall is defined as the number of assigned foreground/true foreground pixels; it shows the rate of exactly how many true foreground pixels are classified as foreground pixels. Precision is defined as the number of true foreground/assigned foreground pixels; it indicates how many pixels are classified as true foreground pixels among the assigned foreground pixels.

(14) $$\begin{array}{cc} \text{Recall} & =\frac{A}{A+C}\text{if}\;A+C>0,\text{otherwise undefined}\\ \text{Precision} & =\frac{A}{A+B}\text{if}\;A+B>0,\text{otherwise undefined} \end{array}$$

High recall or high precision means high performance. However, each performance measure can be misleading when examined alone. For example, a simple algorithm that assigns every pixel to foreground will have a perfect recall of 100%, but an unacceptably low score in terms of precision. Conversely, if a system assigns most of the pixels to background, it will have a high score in terms of precision, but will sacrifice recall to a significant degree. Usually, there is a trade-off between recall and precision; to obtain a high recall usually means sacrificing precision and *vice versa*. Since there is a trade-off between precision and recall, we used the F-measure [21] as another performance measure in order to exactly compare the performance when considering both the precision and recall results simultaneously. The F-measure may be expressed as:

(15) $$F_{\beta }(r,p)=\frac{(\beta^{2}+1)pr}{\beta^{2}p+r}$$

where *β* is a parameter allowing for differential weighting of the precision (*p*) and recall (*r*). When *β* is 1, recall and precision are balanced in such in a way that they have equal weight. The F-measure is maximized when the values of recall and precision are equally high or close. If *β* is set to 1, (15) is denoted as *F_1_*. In this paper, the *F_1_* measure was used to compare the performance of the proposed method with that of the other methods:

(16) $$F_{1}(r,p)=\frac{2pr}{p+r}$$

### 3.2. Experimental Results

#### 3.2.1. Li Dataset

**Figure 6.**
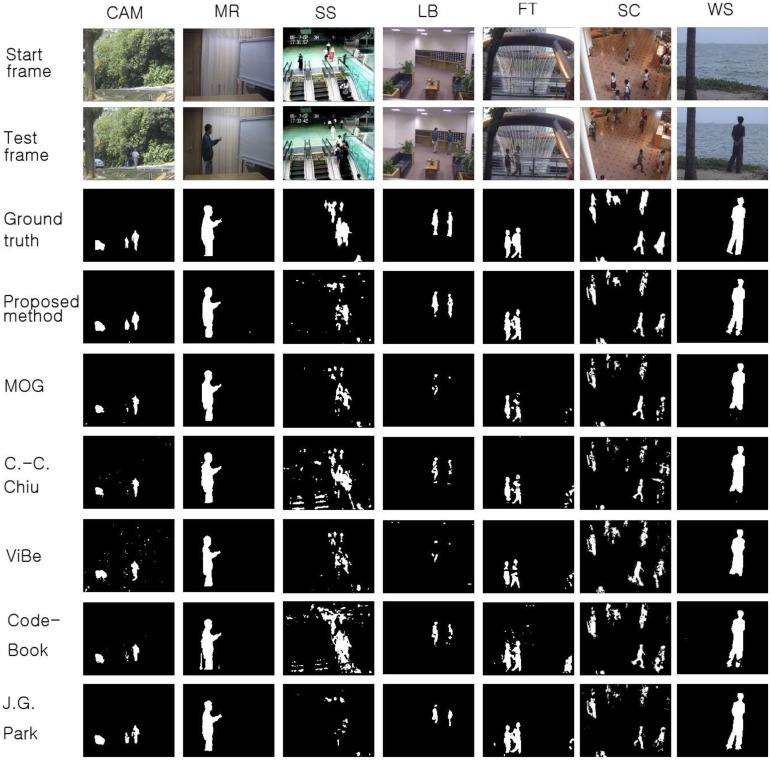
Background subtraction results obtained with the proposed scheme and other methods using the Li dataset. The first frame of each video sequence is shown in the first row, the test frames are displayed in the second row, the ground truth data of the test frames are shown in the third row, and the results obtained with the proposed method are displayed in the fourth row. The results acquired with the other methods are shown in the fifth to eighth rows.

To verify the performance of the proposed method, we used seven video sequences from the Li dataset. The results obtained with the proposed scheme are compared with those from the MOG [4], C.-C. Chiu [5], ViBe [22], and CodeBook [6] methods. The background subtraction results acquired with the proposed method and the other schemes are shown in Figure 6. In the figure, the first frame of each video sequence is shown in the first row, the test frames are displayed in the second row, the ground truth data of the test frames are shown in the third row, and the results obtained with the proposed method are displayed in the fourth row. The results from the other methods are shown in the fifth to eighth rows of Figure 6.

Shown in the first column of Figure 6 is the test sequence CAMPUS (CAM). This sequence has a non-stationary background of a moving tree and contains 1,439 frames with a size of 160 × 128. In the CURTAIN (MR) sequence, there is a waving curtain and sometimes a man appears. In the Escalator (SS) sequence, a moving escalator and many people are shown. The LOBBY (LB) sequence displays an indoor environment; it contains 1,545 frames. This sequence is appropriate for testing sudden illumination changes in an environment. Light is turned off after approximately 500 frames and then turned on after 1,500 frames. The FOUNTAIN (FT) sequence has a non-stationary background with a fountain and a moving object; the sequence contains 522 frames. The ShoppingMall (SC) sequence is also an indoor environment. There are many moving people and numerous shadows appear. Finally, a non-stationary background is tested with the Watersurface (WS) sequence, which contains rippling water.

**Figure 7.**
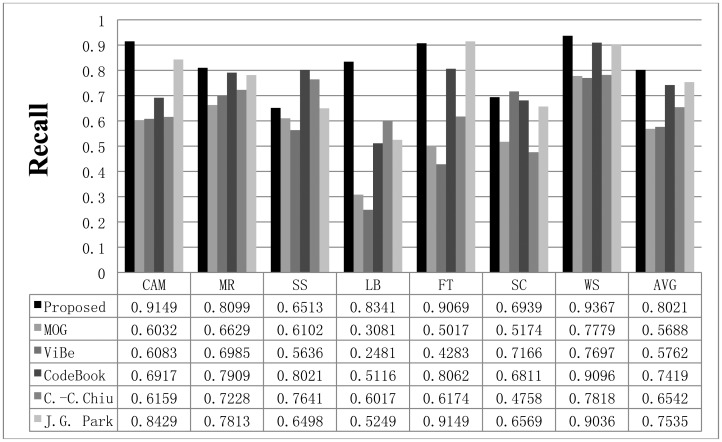
The recall results obtained with the proposed scheme and other methods for the Li dataset. The AVG column represents the average values of the results in all datasets.

**Figure 8.**
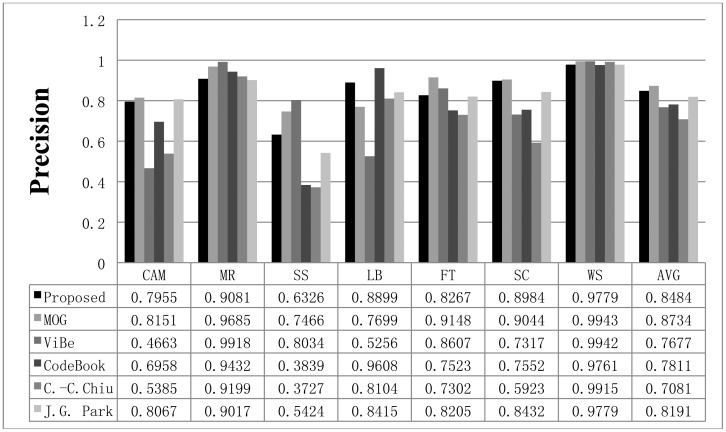
The precision results obtained with the proposed scheme and other methods for the Li dataset. The AVG column represents the average values of the results in all datasets.

**Figure 9.**
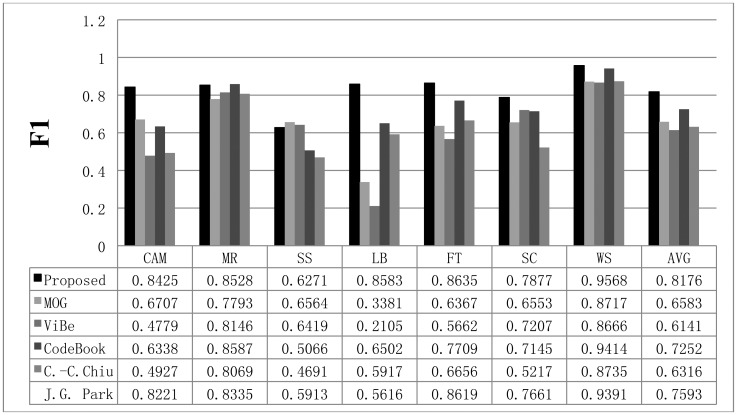
The F-measure results obtained with the proposed scheme and other methods for the Li dataset. The AVG column represents the average values of the results in all datasets.

To compare the performance of the proposed method with that of the other methods, we used parameters presented in the papers detailing the other methods or found appropriate parameters by repeated testing. If a paper detailing one of the other algorithms proposed a parameter set for the image sequence, we used the given parameter value; otherwise, we assumed that the default parameters implemented in the offered algorithm programs are appropriate or we tried to find the best parameters. The Gaussian mixture model was employed and implemented by OpenCV in default mode. The CodeBook algorithm was tested by a program found on the internet [6]. In the program, there are many postprocessing steps such as spot noise removal, blob removal, smoothing, and morphological operations. To compare the proposed scheme with the other methods in the same environment, we ignored the implemented postprocessing steps in the program. In addition, attempts were made to find the best parameters through repeated testing in each sequence. ViBe software [22] was employed to test the ViBe algorithm. Barnich *et al.* [22] showed that the ViBe method generally produces the best results in default mode. In this work, test results were obtained using the ground truths offered from the Li dataset. The recall results acquired with each method are shown in Figure 7. The proposed scheme generally produced better recall results than the other methods. The average value of the recall is also better with the proposed method. The precision results obtained with all methods are shown in Figure 8, while the F1 results are displayed in Figure 9. Regarding the precision results, the proposed method generally exhibits good performance. The F1 results show the general performance when considering precision and recall. The obtained results show that the proposed method is very effective in extracting the foreground. The proposed method has a better result in LB sequence than other methods, because it can effectively adapt to the changing environment.

#### 3.2.2. Wallflower Dataset

**Figure 10.**
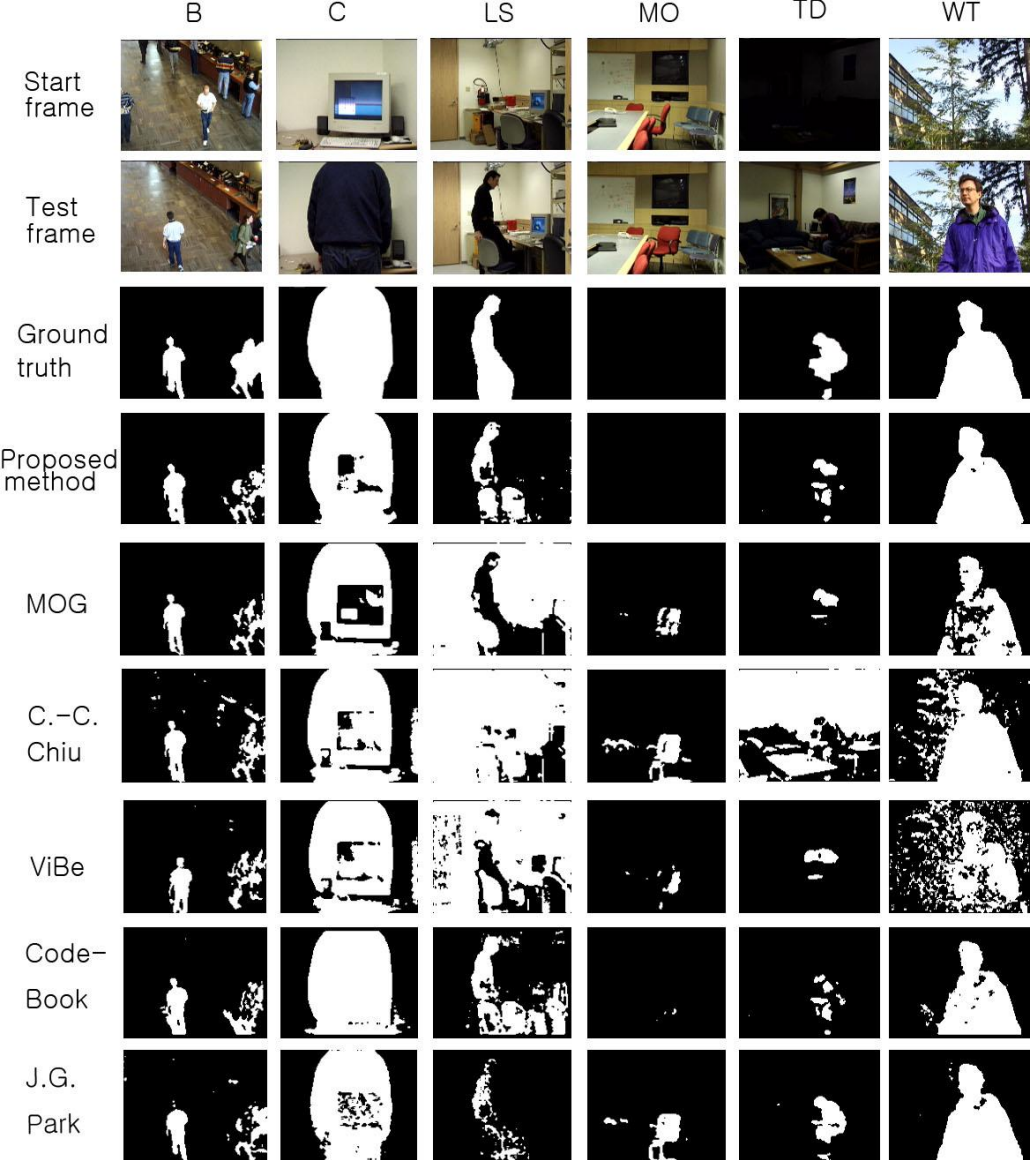
Background subtraction results obtained with the proposed scheme and other methods using the Wallflower dataset. The first frame of each video sequence is shown in the first row, test frames are displayed in the second row, ground truth data for the test frames are shown in the third row, and the results obtained with the proposed method are displayed in the fourth row. The results obtained with the other methods are shown in the fifth to eighth rows.

We used six sequences in the Wallflower dataset to test our method. The first sequence is BOOTSTRAP (B), which contains many moving people and numerous shadows. If the updating speed for the background is too fast or the threshold is too high, people at the desk can be classified as part of the background. In the sequence, the proposed algorithm is able to effectively eliminate the shadows, while the other methods sometimes cannot reduce errors. The second sequence is CAMOUFLAGE (C). In this sequence, the codebook method yields the best result. While our method has a lower recall than codebook, it exhibits higher precision than codebook and the other methods. We can confirm that our method is able to adapt to sudden environment changes by applying the LIGHTSWITCH (LS) sequence containing 2,714 frames. In the sequence, the light is turned off after 812 frames and then turned on again at frame 1,854. The sequence MOVEOBJECT (MO) contains 1,745 frames with a moving object. MOVEOBJECT is appropriate to test the adaptability of the background model. When the chair is moved at frame 888, it should become part of the background after a suitable period of time. The recall, precision, and F1 results for the MO sequence are not displayed here. However, the adaptability of each background modeling method is shown in Figure 10. The TIMEOFDAY (TD) sequence contains 5,889 frames. As time progresses, the image gradually becomes brighter or darker. Finally, the WAVINGTREES (WT) sequence is a non-stationary background with a waving tree; it contains 287 frames.

**Figure 11.**
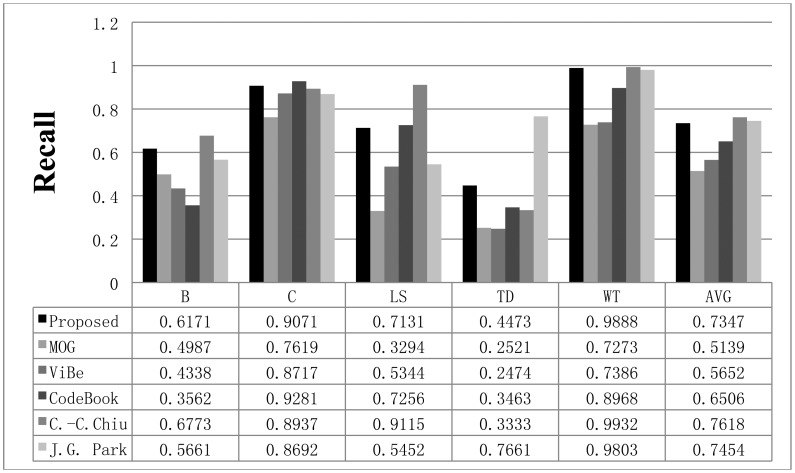
The recall results obtained with the proposed scheme and other methods for the Wallflower dataset. The AVG column represents the average values of the results in all datasets.

**Figure 12.**
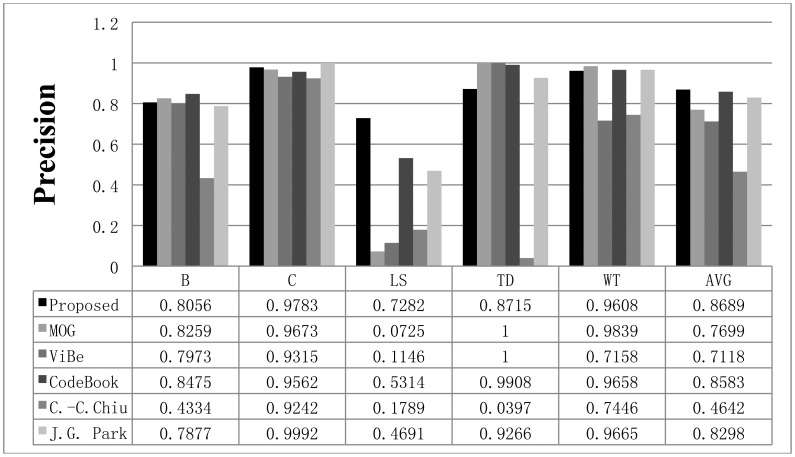
The precision results obtained with the proposed scheme and other methods for the Wallflower dataset. The AVG column represents the average values of the results in all datasets.

**Figure 13.**
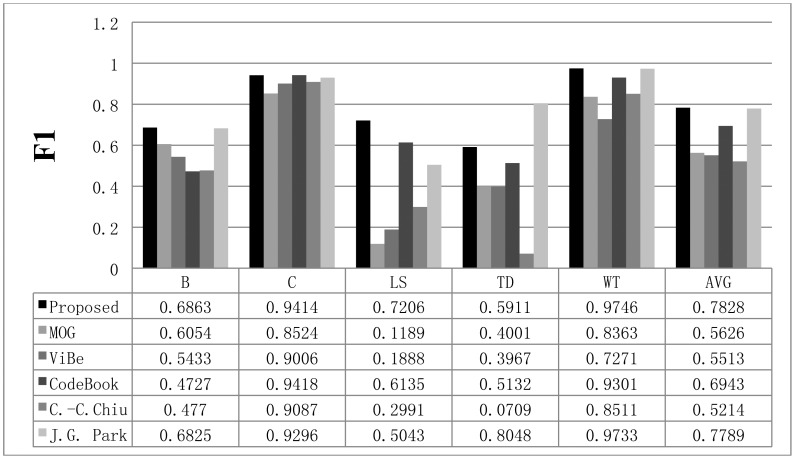
The F1 results obtained with the proposed scheme and other methods for the Wallflower dataset. The AVG column represents the average values of the results in all datasets.

The recall results obtained with all methods are shown in Figure 11, while the precision results are displayed in Figure 12. The F1 results are also shown in Figure 13. When compared to the other methods, the proposed scheme was found to be more robust to sudden illumination changes, such as in the LS sequence. We can also confirm that the proposed method is generally better than the other methods in the Wallflower dataset. In the TD sequence, if the input sequences are continuously changing, the proposed method occasionally misclassifies the foreground as the background, because it uses change rates of the sequence to extract foreground. We did not include the MO results in the figures. Because there are no objects in the ground truth of the MO sequence, the recall and precision are undefined, but Figure 10 shows that our method effectively adapts to the changed environments and classifies the moved object as background after some time (according to the updating rate). In real environments, this character is very important, because background environments can be frequently changed. For example, when a car leaves in some large parking lots (here means many parking spaces), the blanked parking space is detected as moving object at first and be considered background after some time.

#### 3.2.3. Parameter Set and Computational Cost

In this work, we investigated the effects of different values of *N_d_*. To simplify the algorithm, *N_d_* was selected to have same values in each dimension.

If the number of *N_d_* is increased, we can get more exact result. But, the performance was almost constant for *N_d_* ≥ 60. Large number of *N* means more memory burden and more computational cost required. So, the *N_d_* was selected as 60 in the experiments.

Since the proposed method used histograms instead of density estimation and previously calculated the Gaussian values according to distance to avoid repeating such a complex calculation, the process of the proposed method is simplified. Also, because the proposed method does not use other complex approaches such as calculating gradient information or thresholds considering whole pixel values, it is much faster than the KDE method and Park [10]. To compare the performance, the algorithms were implemented using the C programming language on a 2.53 GHz CPU with 2 GB of RAM. Compared to the proposed method, Park [10] takes too much time to obtain KDE background model. When we tested the time of modeling background of Park [10] with 100 frames of 160 × 128 pixels, it takes about 15.7 s, while the proposed method does not need such a learning time. The classification time of the proposed method was about 41.6 frames per second, while the Park [9] was about 31.6 frames per second.

## 4. Conclusions

An adaptive background subtraction method based on kernel density estimation was presented. The background is modeled as a probabilistic model by kernel density estimation. To reduce the computational complexity and memory requirements, we modified the original kernel density estimation method and applied histogram approximation and modified the updating method. This method automatically adapts to the environment as time progresses and it can reduce the complexity compared with original KDE approach method. In the initial stage, the proposed method could not correctly extract foreground, because the moving object and passing space of the moving object can be classified as background, so the background needs to re-update fast in the initial stage. The updating process should be stabilized as time goes on, so we applied a sigmoid function to control the learning rate according to the environment. When we set *β* as 100, *Gain* as 300 and *λ* to be 20 in Equation (4) in our experiment, the background model was stabilized around after 100 frames. This method makes up for a drawback of initializing background model at first frame. Consequently, the algorithm can quickly adapt to a given environment. The proposed method used the difference values between an input image and the background model. The average mean value of the difference was employed to extract the foreground and allow for effective adaption to the environment. The recall, precision, and F-measure were used to evaluate the performance. The proposed method obtained generally high result in most of the sequences than other methods. A comparison of the proposed algorithm with other methods revealed that the proposed method is very effective in extracting the foreground in various environments.

## Figures and Tables

**Figure 14.**
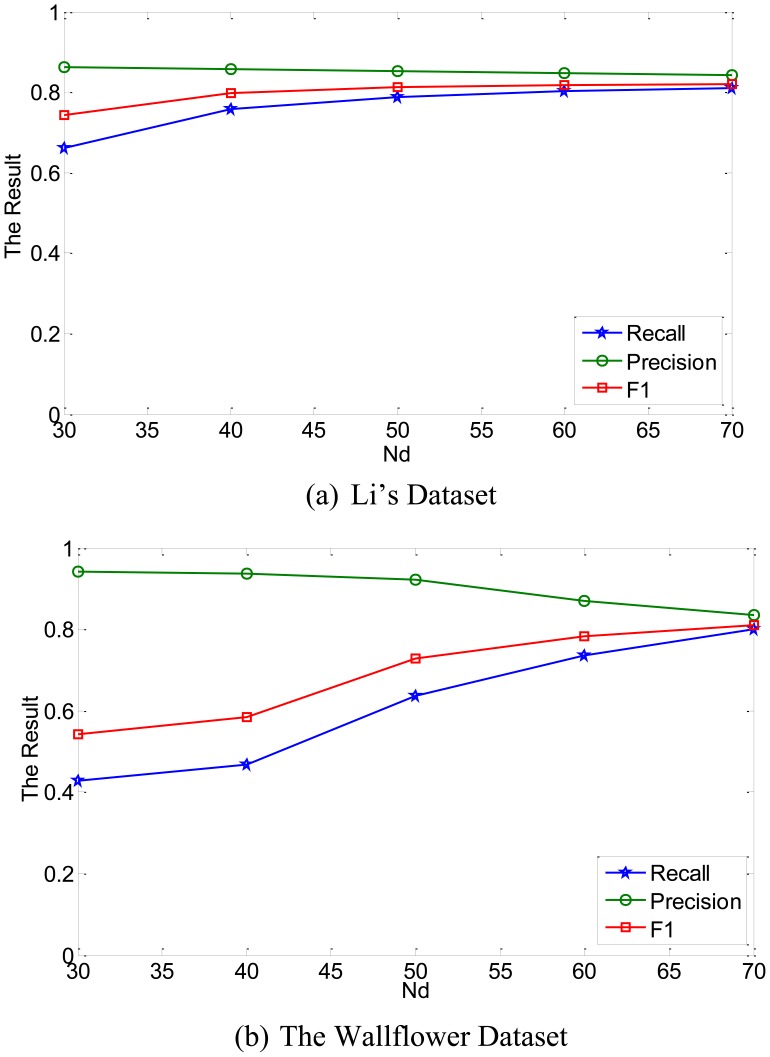
The evaluation performance as a function of *N_d_*.

**Table 1.** A contingency table.

	**Foreground is correct**	**Background is correct**
**Assigned foreground**	A	B
**Assigned background**	C	D
